# F239. THE ROLE OF DANCE/MOVEMENT THERAPY IN THE TREATMENT OF NEGATIVE SYNDROME AND PSYCHOSOCIAL FUNCTIONING OF PATIENTS WITH SCHIZOPHRENIA: RESULT FROM A PILOT MIXED METHODS INTERVENTION STUDY WITH EXPLANATORY INTENT

**DOI:** 10.1093/schbul/sby017.770

**Published:** 2018-04-01

**Authors:** Karolina Bryl

**Affiliations:** Drexel University, College of Nursing and Health Professions

## Abstract

**Background:**

Optimizing psychosocial functioning and overall well-being by reducing the severity of negative symptoms are important outcomes for individuals with schizophrenia. Movement-based therapeutic approaches are uniquely capable of addressing the non-verbal nature of negative symptoms. Dance/Movement Therapy (DMT), a promising treatment for mental health conditions such as schizophrenia, has been found to reduce the occurrence and severity of negative symptoms and to have a positive impact on the psychosocial functioning. Although preliminary findings suggest DMT as a treatment intervention, limited research and inconclusive findings preclude generalizations and more research is needed. We aimed to examine the treatment effects of a 10-week (20 sessions) group DMT treatment program.

**Methods:**

We employed a mixed methods intervention design with explanatory intent, in which a randomized controlled trial is followed by semi-structured exit interviews. Thirty-one severely ill individuals diagnosed with schizophrenia participated in the RCT that used a two-arm parallel group design to assess and show the difference between patients receiving standard care (SC) and patients receiving standard care plus DMT on measures of negative symptoms (as primary outcome; PANSS, BNSS) and psychosocial functioning (as secondary outcomes; WHO-DAS 2.0, SDS). Quantitative measures were taken pre and post- intervention. Participants who participated in a minimum of 50% of DMT sessions (n=15) were invited to an exit interview. This criterion was also used to analyze quantitative data, leaving n=28 for quantitative analysis.

**Results:**

All participants in both groups (n=31) completed the study. Because of such a small sample size (n=28) and a pilot nature of the study we were restricted to use descriptive statistics.

The quantitative data suggest that DMT and SC were not equally effective in enhancing primary outcomes. Analysis of the PANSS mean score changes showed a slight increase in the negative symptom in the DMT from 28.33 ± 4.76 to 29.00 ± 4.10, and slight decrease in the SC from 28.92 ± 5.72 to 27.08 ± 5.64. BNSS scores indicate that both groups improved. SC participants reported grater reduction on BNSS overal score from 53.31 ± 11.48 to 47.77 ± 8.10 in comparison to DMT from 53.07 ± 7.27 to 51.93 ± 6.18. However, DMT participants reported reduction of symptoms in distress, antisocial activity, avolition and verbal expression.

Analysis of WHO-DAS suggests that DMT was effective in reduction of disability severity compared to SC. DMT participants reported grater improvement in cognition, mobility, self-care, and getting along. Both groups reported reduction of the impact of difficulties on daily functioning on SDS, however DMT participants reported a greater reduction in days during which they were completely unable to perform or had to limit their usual activities or work due to symptoms. In the SC, the results suggest a reduction in the number of days lost and days of lower productivity.

Qualitative findings identified participants’ experiences and the most important themes related to benefits of the DMT intervention: enhanced activation, motivation, socialization, and self-awareness.

**Discussion:**

Results of this study contribute to knowledge about body-based interventions for schizophrenia and indicate that DMT had an effect on participants psychosocial functioning and coping with negative symptoms. Integration of quantitative and qualitative data provides a wider perspective by gaining a better understanding of the treatment outcomes and explaining inconclusive results. Findings of this study set the stage for larger fully powered research, examining intervention methods and procedures, as well as treatment effects, more thoroughly.

